# Study on Microstructure and Mechanical Properties of Core–Shell-Structured Ti@Ti_x_N Reinforced Al Composite Prepared by Pressure Infiltration

**DOI:** 10.3390/ma18061200

**Published:** 2025-03-07

**Authors:** Yixiao Xia, Zhiyu Sun, Ping Zhu, Juanrui Hu, Leilei Hao, Yun Liu, Boyu Ju, Guoqin Chen, Wenshu Yang

**Affiliations:** 1 School of Materials Science and Engineering, Harbin Institute of Technology, Harbin 150001, China; etialxia@icloud.com (Y.X.); 19504565166@163.com (Z.S.); 18846450834@163.com (P.Z.); yws001003@163.com (W.Y.); 2 State Key Laboratory of Precision Welding & Joining of Materials and Structures, Harbin Institute of Technology, Harbin 150001, China; 3 Beijing Institute of Aerospace Control Devices, Beijing 100080, China; 18610697362@163.com (J.H.); 13161880493@163.com (L.H.); 13621067604@163.com (Y.L.); 4 Zhengzhou Research Institute, Harbin Institute of Technology, Zhengzhou 450018, China

**Keywords:** Al matrix composite, mechanical properties, interface design, core–shell structure

## Abstract

In this research, a nitrogenized shell layer was formed on the surface of Ti powder in a high-temperature N_2_ environment, resulting in core–shell-structured Ti@Ti_x_N powder. Using this as a reinforcement, Ti@Ti_x_N/Al composite was successfully designed and fabricated via pressure infiltration method. The Ti_x_N layer consists of a double-layered spherical shell structure, with TiN as the outer layer and Ti_2_N as the inner layer. After the composite was fabricated, no intermetallic compounds between Ti and Al were observed at the interface, as the Ti_x_N layer effectively prevented the reaction between Ti and Al. The tensile strength, yield strength, and elongation of the Ti@Ti_x_N/Al composite were 173 ± 7.7 MPa, 115 ± 8.1 MPa, and 7.5 ± 0.55%, respectively. Both the strength and hardness were significantly improved compared to the pure Al matrix. Observations of the tensile fracture surface revealed severe interfacial debonding at the interface, and the reinforcement did not exhibit significant coordinated deformation with the matrix. This suggests that future research could focus on strengthening the matrix by adding alloying elements and improving the interfacial bonding to enhance the performance of the composite.

## 1. Introduction

Aluminum matrix composites (AMCs) have attracted significant attention due to their excellent properties such as high strength, high stiffness, and low density. Traditional ceramic-reinforced AMCs have been extensively studied, such as SiC/Al [1,2], Al_2_O_3_/Al [3,4], B_4_C/Al [5], and AlN/Al [6]. For AMCs that require high modulus, a high content of reinforcements is necessary, which leads to a sharp decline in the plasticity of AMCs [7], thereby increasing the difficulty of processing such AMCs. In recent years, metal particle-reinforced AMCs have become a research hotspot, with reinforcements including metallic glass [8], Al particles [9], Mg particles [10], and Be particles [11,12], among others. The characteristic of these AMCs is that the reinforcement particles can undergo coordinated deformation with the matrix, thereby maintaining high plasticity and processability even at high reinforcement content, and achieving high strength through deformation. Tian et al. [13] conducted extrusion on a 20 vol.% Ca/Al composite, and the filament thickness of the Ca reinforcement decreased with increasing deformation. Meanwhile, as the true strain increased from 1.93 to 12.91, the ultimate tensile strength increased from 122 MPa to 476 MPa.

Ti has various oxides and nitrides. Ti oxides include black titania and various Magnéli phases [6]. Ti nitrides mainly include TiN and Ti_2_N [14]. When preparing Ti nitrides using Ti powder as the raw material, nitrogen sources are typically N_2_ or NH_3_, and the methods include direct nitridation [15], combustion synthesis [16], ball milling [17], and electrolysis [18]. Among these, the direct nitridation process is the simplest. According to the research by Wriedt et al. [14], due to the nitrogen concentration gradient, TiN with a higher nitrogen content forms on the outer layer of the Ti powder, while the inner layer consists of Ti_2_N with a relatively lower nitrogen content. When TiN is heated in air, it reacts with O_2_ to form Ti oxides, such as the rutile-TiO_2_ phase [19,20]. The research by Hou et al. [21] shows that the oxidation of TiN begins at a temperature of 800 K and is influenced by the partial pressure of O_2_ and the heating rate. The higher the O_2_ partial pressure and the lower the heating rate, the faster the oxidation rate.

Ti/Al composites have also become a research hotspot in recent years due to their low density, low cost, high strength, excellent corrosion resistance, and high-temperature resistance [22,23,24,25]. However, the interfacial bonding quality between Ti and Al significantly affects the mechanical properties of Ti/Al composites [26]. Wang et al. [27] found that, after long-term annealing, a thick and brittle TiAl_3_ intermetallic compound layer formed at the interface of Ti_6_Al_4_V/AA6061 composite, which had a significant impact on the performance of the composite. Yu et al. [28] demonstrated that an excessive intermetallic compound layer reduced the shear strength of the Al/Ti interface. To improve the interfacial bonding of Ti/Al, Liu et al. [29] introduced Mg and Si alloying elements into the matrix, which improved the interface constraint potency and dislocation hardening capability. Xiu et al. [30] applied a TiN coating on the surface of Ti fibers to prevent direct contact between Ti and Al, thereby inhibiting the formation of the brittle TiAl_3_ phase caused by the reaction between Ti and Al.

Currently, the methods for fabricating Ti/Al laminated composites include ultrasonic additive manufacturing [31], hot press bonding [32], hot rolling bonding [33], and cold rolling bonding [34]. For the preparation of Ti powder-reinforced AMCs, ball milling is typically used to mix the powders [35,36], and spark plasma sintering is employed to fabricate the composites [25]. However, as mentioned earlier, these methods face challenges in addressing the interfacial bonding issues between Ti and Al. The pressure infiltration method used in this study is a solid–liquid bonding fabrication technique, where liquid Al solidifies on the surface of the solid reinforcement, naturally forming good mechanical bonding. This method has been successfully used to fabricate various composites [37,38,39,40].

Building on the previous work of our team (Xiu et al. [30,41]), this study formed a nitride layer on the surface of Ti powder in a high-temperature N_2_ environment, resulting in core–shell-structured Ti@Ti_x_N powder. Using Ti@Ti_x_N powder as the reinforcement, Ti@Ti_x_N/Al composite was fabricated via the pressure infiltration method, with the Ti_x_N layer effectively preventing harmful reactions between Ti and Al. The microstructure and mechanical properties of the Ti@Ti_x_N/Al composite were subsequently investigated.

## 2. Materials and Methods

### 2.1. Raw Materials

**Figure 1:**
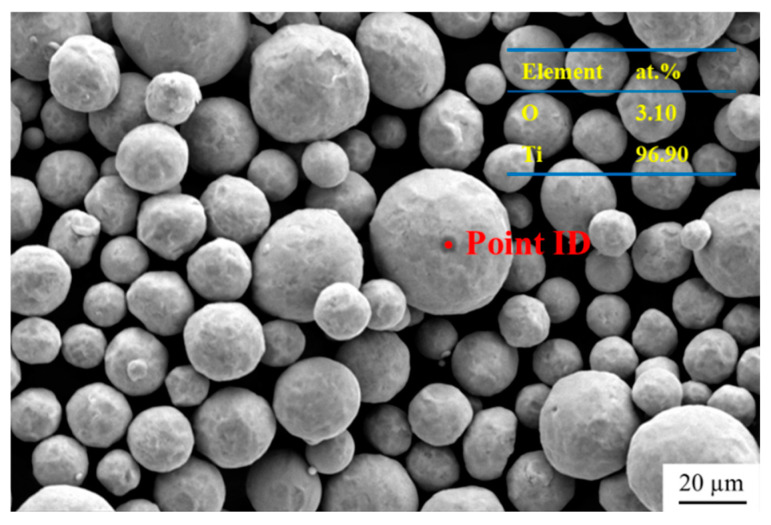
The morphology and EDS analysis of Ti powder.

The Ti powder used in this study was high-purity titanium powder (TA1), supplied by Sino-Euro Materials Technologies of Xi’an Corp. Ltd., Xi’an, China. The morphology and energy dispersive spectrometer (EDS) point analysis are shown in Figure 1. The powder is roughly spherical, with a particle size distribution of 15–53 µm. It can be observed that the Ti powder has very high purity and a very low degree of oxidation. The matrix was pure Al, provided by Northeast Light Alloy Corp. Ltd., Harbin, China. The purity of N_2_ gas used in this work is 99.99%, provided by Liming Gas Corp. Ltd., Harbin, China.

### 2.2. Fabrication Process

**Figure 2:**
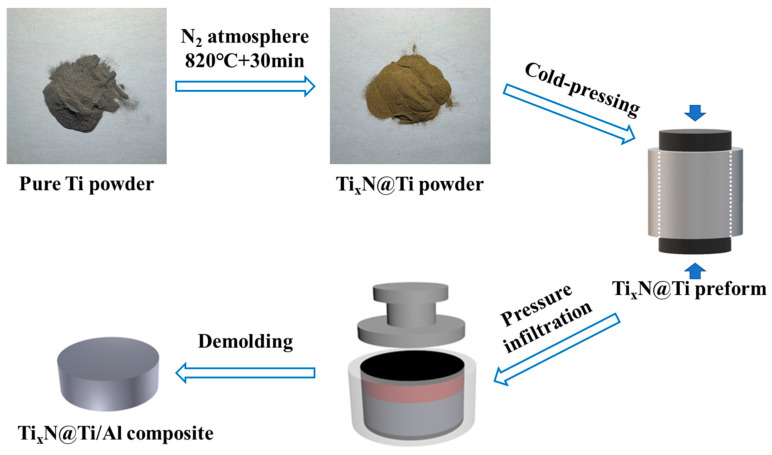
Schematic preparation process of Ti@Ti_x_N/Al composite.

The schematic diagram of the powder nitriding and composite fabrication process is shown in Figure 2. The nitriding process of Ti powder was processed in a nitriding furnace. Before nitriding, the chamber needed to be cleaned. The specific steps are as follows: first, the chamber was evacuated to −0.1 MPa (relative to atmospheric pressure), then N_2_ was introduced until the chamber pressure reached 0 MPa. This evacuation and refilling process was repeated three times. Finally, N_2_ was introduced until the chamber pressure reached 0 MPa. The temperature was then increased to 820 °C at a rate of 50 °C/min and held for 30 min. During the nitriding process, N_2_ was continuously supplied to the chamber at a flow rate of 200 mL/min. After nitriding, the powder was cooled to room temperature in the furnace, resulting in Ti@Ti_x_N powder with a golden-yellow surface. Subsequently, the Ti@Ti_x_N powder was loaded into a mold and cold-pressed into a preform. During cold pressing, the pressure was kept below 50 MPa to prevent deformation of the powder. The preform was then held at 580 °C for 1 h in an N_2_ environment, while the Al ingot was melted at 780 °C. The liquid aluminum was then poured into the preform mold and kept under a pressure of 10 MPa for 5 min to ensure complete infiltration of the aluminum into the preform. Finally, the 60 vol.% Ti@Ti_x_N/Al composite was obtained by cooling and demolding.

### 2.3. Characterization

The morphology of Ti and Ti@Ti_x_N powders and the microstructure of Ti@Ti_x_N/Al composite were characterized by a Merlin Compact scanning electron microscope (SEM) (Carl Zeiss, Oberkochen, Germany) in secondary electron (SE) mode equipped with EDS. Before SEM microstructure observation, the Ti@Ti_x_N/Al composite was polished using SiC sandpaper with grits of 200, 600, 1000, 1500, 2000, 3000, and 5000, then polished using a 0.5 µm diamond abrasive.

The XRD test was used to characterize the phase composition of Ti and Ti@Ti_x_N powders and Ti@Ti_x_N/Al composite. The XRD test was performed on Rigaku D/max-rB diffractometer (Rigaku, Tokyo, Japan) with the scanning range between 20° and 90° at the scanning speed of 2°/min.

Raman spectroscopy (Zolix Omni λ500i, Beijing Zhuoli Hanguang Instrument Co., Ltd., Beijing, China) was used to characterize the compound analysis of Ti powder, Ti@Ti_x_N powder, and annealed Ti@Ti_x_N powder. The Raman tests were conducted with a laser wavelength of 532 nm, an aperture of 600, and a testing range of 100–700 cm^−1^.

**Figure 3:**
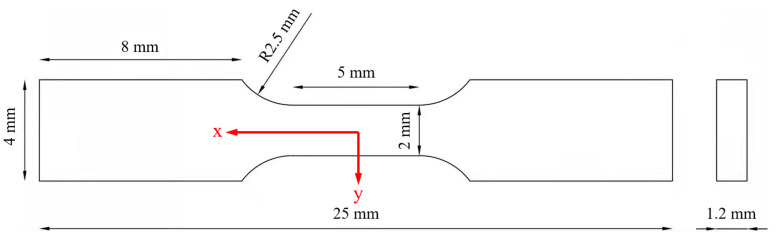
Dimensions of tensile sample.

The tensile tests were conducted using an Instron 5569 universal electrical testing machine. The dimensions of the tensile specimens are shown in Figure 3, where the tensile direction is defined as the *x*-axis, and the direction perpendicular to the thickness is defined as the *z*-axis. The test speed was 0.5 mm/min. Before conducting the mechanical property tests, the Ti@Ti_x_N/Al composite was fully annealed. The Brinell hardness test was performed using a Brinell hardness tester (LHBS-3000MD, YunCe, Kunshan, China). The sample size was 10 × 10 × 3 mm. To improve the reliability of the data, at least five samples were tested for each group.

## 3. Results and Discussion

### 3.1. Morphology and Phase Composition of Powders

**Figure 4:**
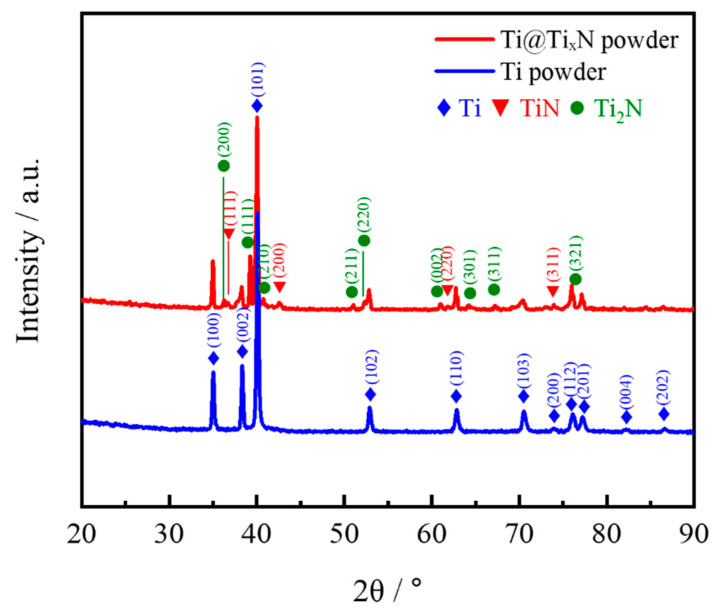
The XRD patterns of the Ti and Ti@Ti_x_N powders.

**Figure 5:**
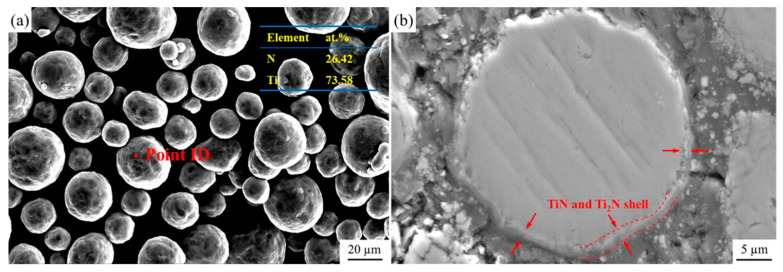
(**a**) Morphology and (**b**) transversal surface of Ti@Ti_x_N powder by SEM.

The XRD patterns of Ti and Ti@Ti_x_N powders are shown in Figure 4. The Ti powder exhibits very high purity, with no impurities detected. After nitriding treatment, two types of nitrides, TiN and Ti_2_N, appeared in the Ti powder. As shown in Figure 5a, the microstructure of the Ti@Ti_x_N powder was observed. Compared to pure Ti powder, the surface smoothness of the nitrided powder decreased significantly. EDS analysis revealed that the surface layer of the powder contained approximately 26.42 at.% nitrogen. The Ti@Ti_x_N powder was cross-sectioned, as shown in Figure 5b, where a distinct nitride layer forming a spherical shell can be observed, indicated by the red arrows. According to the research by Wriedt et al. [14], nitrogen atoms diffuse into the interior of Ti particles, forming a Ti(N) solid solution shell. When the nitrogen atom concentration in the solid solution reaches 33 at.%, Ti_2_N is formed. As the nitrogen concentration further increases to 37 at.%, the TiN phase is generated. This explains the two nitrides detected in the XRD patterns, with TiN located on the outer side of the shell where the nitrogen concentration is higher.

**Figure 6:**
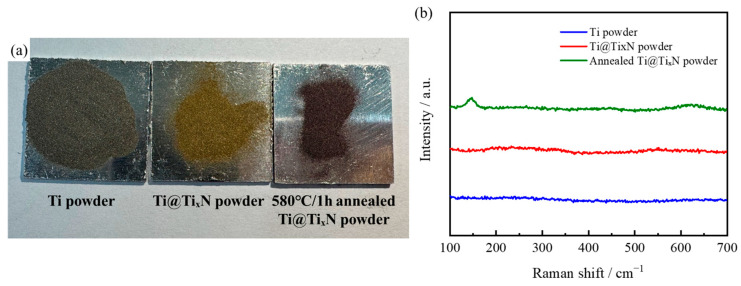
(**a**) Macroscopic morphologies and (**b**) Raman scattering spectra of powders.

As shown in Figure 6a, the macroscopic morphologies of Ti powder, Ti@Ti_x_N powder, and annealed Ti@Ti_x_N powder are presented. The Ti powder appears silver-gray, while the Ti@Ti_x_N powder exhibits a golden color due to the TiN on its surface. We performed an annealing treatment on the Ti@Ti_x_N powder in an air environment at 580 °C for 1 h, resulting in the powder turning deep red and black. Subsequently, Raman analysis was performed on the three types of powders, and the results are shown in Figure 6b. The blue curve represents the results for Ti powder, and the red curve represents the results for Ti@Ti_x_N powder. Neither curve shows any significant signals, indicating that no obvious oxidation occurred in the Ti powder and Ti@Ti_x_N powder, which is consistent with the XRD results and reference [20]. In contrast, the annealed Ti@Ti_x_N powder was also tested, and a prominent peak was detected around 145 cm^−1^, corresponding to black TiO_2_ [42]. Additionally, a peak was detected around 600 cm^−1^, corresponding to the rutile-TiO_2_ phase [20]. This explains the deep red and black appearance of the annealed Ti@Ti_x_N powder. Furthermore, since annealing the Ti@Ti_x_N powder at 580 °C can lead to significant oxidation on the surface, a nitrogen atmosphere was used for protection during the holding process of the preform before preparing the composites.

### 3.2. Morphology and Phase Composition of Composite

**Figure 7:**
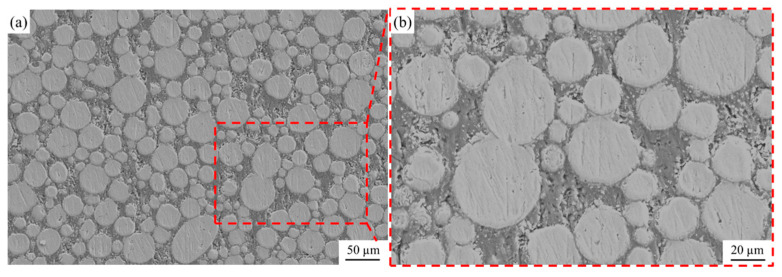
SEM characterization of Ti@Ti_x_N/Al composite; (**b**) is the local amplification of (**a**).

**Figure 8:**
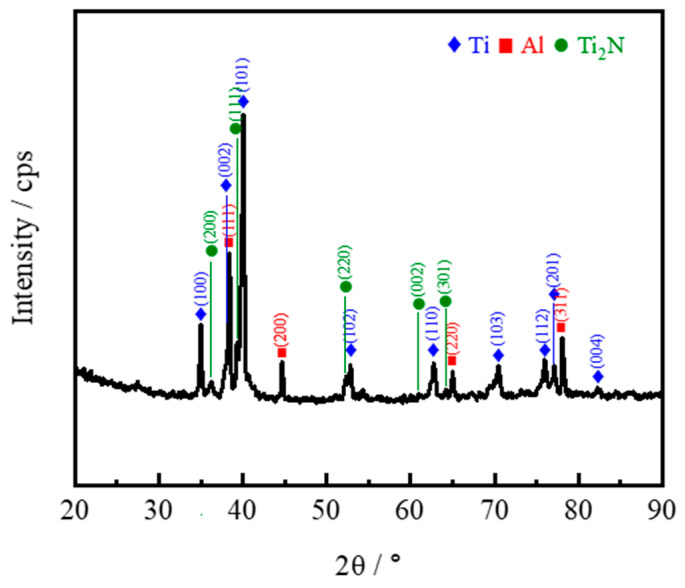
XRD pattern of Ti@Ti_x_N/Al composite.

Figure 7 shows the SEM images of the Ti@Ti_x_N/Al composite. The Ti@Ti_x_N reinforcements are uniformly distributed within the matrix, and the interface between the matrix and the reinforcements remains intact and clean, indicating good composite quality during the fabrication process. No obvious interfacial reaction layer was observed at the interface, which is further confirmed by the XRD pattern of the Ti@Ti_x_N/Al composite (Figure 8). The phases present in the composite are mainly Ti, Al, and Ti_2_N, with no intermediate compounds of Ti and Al detected. This demonstrates that the Ti_x_N shell successfully prevented the reaction between Ti and Al. It is worth noting that in the XRD pattern of Figure 4, the peaks of TiN are very small, which indicates that the content of TiN is relatively low. When the Ti@Ti_x_N powder is prepared into a composite, the relative content of TiN further decreases. This results in the disappearance of the TiN peaks in the XRD pattern.

### 3.3. Mechanical Properties and Fracture Analysis

**Figure 9:**
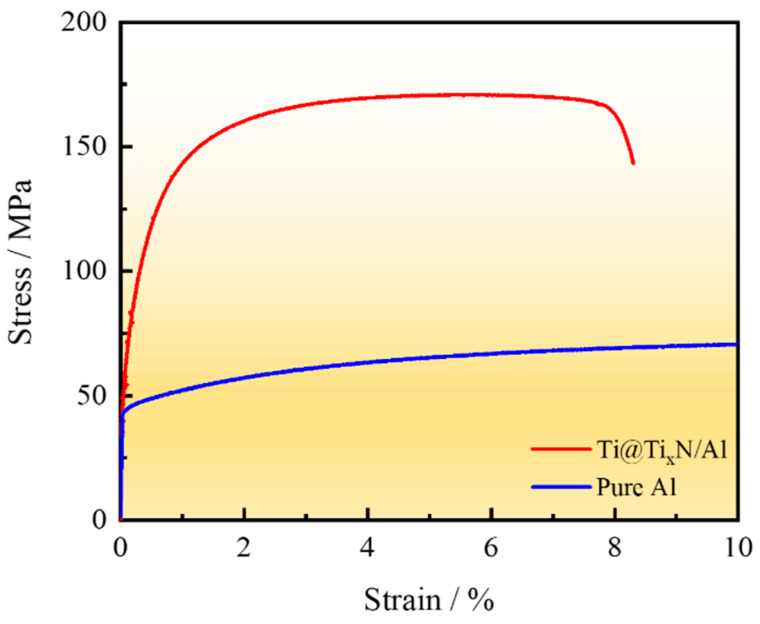
Strain–stress curve of Ti@Ti_x_N/Al composite and matrix.

Figure 9 shows the tensile curves of the Ti@Ti_x_N/Al composite and the matrix. Table 1 lists the mechanical properties of the matrix and the Ti@Ti_x_N/Al composite. The tensile strength and yield strength of the Ti@Ti_x_N/Al composite are 173 ± 7.7 MPa and 115 ± 8.1 MPa, respectively, with an elongation of 7.5 ± 0.55% and a Brinell hardness of 71.6 ± 8.4 HB. In comparison, the pure Al matrix has a tensile strength of 74 MPa, a yield strength of 44 MPa, and a Brinell hardness of 20.1 ± 0.2 HB. While the plasticity of the Ti@Ti_x_N/Al composite is reduced compared to the matrix, its tensile strength, yield strength, and Brinell hardness are significantly improved.

**Figure 10:**
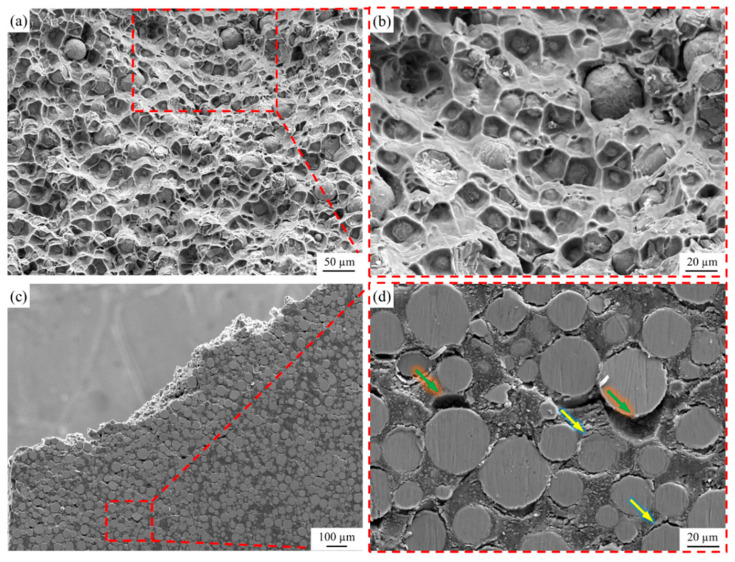
Fracture surfaces of Ti@Ti_x_N/Al composite. (**a**,**b**) *x* direction, (**c**,**d**) *z* direction.

The tensile fracture surface of the Ti@Ti_x_N/Al composite was observed, as shown in Figure 10. Figure 10a,b show the fracture surface observed from the x direction. A large number of exposed Ti@Ti_x_N particles can be seen in the dimples of the fractured Al matrix, indicating that the primary fracture mechanism of the composite is interfacial debonding between the reinforcement and the matrix. Figure 10c,d show the fracture surface observed from the z direction, where it can be seen that cracks mainly propagate along the interface between the reinforcement particles and the matrix. Notably, due to the good plasticity of the Al matrix, even after interfacial debonding, significant plastic deformation can occur without immediate fracture, as indicated by the green arrows in Figure 10d. This is one of the reasons why the Ti@Ti_x_N/Al composite is able to retain a certain level of plasticity. Additionally, some cracks were observed to propagate across the interface into the reinforcement particles, as indicated by the yellow arrows in Figure 10d. This suggests that, in localized regions, the Ti@Ti_x_N particles play a significant load-bearing role.

The main reason for the significant interfacial debonding is the relatively weak interfacial bonding strength between the Al matrix and TiN. Yadav et al. [43] found that when the bonding between TiN and the Al matrix is N-terminated, strong bonding interactions are formed between Al and N atoms, and the shear fracture occurs within the Al matrix. Furthermore, during the tensile process, due to the low strength of the pure Al matrix, it cannot generate sufficient stress to deform the Ti@Ti_x_N particles. As a result, there is no significant coordinated deformation between the Ti@Ti_x_N particles and the Al matrix. This is a key factor limiting the strength and plasticity of the Ti@Ti_x_N/Al composite.

Currently, the main Ti/Al composites are laminated Ti/Al composites [24,31,32,44]. During the preparation process, rolling is often used to enhance the interfacial bonding strength [22,34,45], which also alters the microstructure of Ti and Al compared to the as-cast state, thereby improving the strength and ductility of the composites. This provides insights for improving the performance of Ti@Ti_x_N/Al composite in the future, such as applying hot extrusion, hot rolling, and other thermomechanical processes to the Ti@Ti_x_N/Al composite. In other studies, higher-strength Ti alloys and Al alloys were directly used as raw materials to achieve Ti/Al composites with higher strength [26,27].

Therefore, our future work will focus on hot working, as well as selecting appropriate alloying elements to strengthen the Al matrix and Ti powder while promoting the formation of strong chemical bonds between the Al matrix and the reinforcement to improve interfacial bonding strength.

## 4. Conclusions

In this study, pure Ti powder was used as the raw material, and N_2_ gas reacted with Ti powder at high temperatures to form a nitride shell on the surface of the Ti powder. Subsequently, core–shell-structured Ti@Ti_x_N powder was used as the reinforcement, and Ti@Ti_x_N/Al composite was fabricated via the pressure infiltration method. The microstructure and mechanical properties of the composite were investigated. The main conclusions are as follows:

1. The Ti_x_N shell formed by the reaction between N_2_ gas and Ti powder consists of TiN and Ti_2_N, with TiN in the outer layer and Ti_2_N in the inner layer.
2. The Ti_x_N shell prevented the reaction between Ti and Al, and no intermediate phases of Ti and Al were observed in the composite.
3. The tensile strength, yield strength, and elongation of the Ti@Ti_x_N/Al composite are 173 ± 7.7 MPa, 115 ± 8.1 MPa, and 7.5 ± 0.55%, respectively, with a hardness of 71.6 ± 8.4 HB. Compared to the pure Al matrix, these properties are significantly improved, and the Ti@Ti_x_N reinforcement played a certain load-bearing role.
4. The fracture mode of the Ti@Ti_x_N/Al composite is primarily interfacial debonding, while the plasticity of pure Al maintained the overall plasticity of the composite.

## Figures and Tables

**Table 1:** Mechanical properties of Al matrix and Ti@Ti_x_N/Al composite.

Material	Brinell Hardness/HB	Tensile Strength/MPa	Yield Strength/MPa	Elongation/%
Pure Al	20.1 ± 0.2	74	44	38.7%
Ti@Ti_x_N/Al composite	71.6 ± 8.4	173 ± 7.7	115 ± 8.1	7.5 ± 0.55

## Data Availability

The data presented in this study are available on request from the corresponding authors due to privacy.
